# Supplementary material for: Prostate cancer genetic risk and associated aggressive disease in men of African ancestry
Source: Nat Commun. 2023 Dec 5;14:8037. doi: 10.1038/s41467-023-43726-w (PMC10697980; doi:10.1038/s41467-023-43726-w)
Supplement: Supplementary file 1 — Supplementary Information [file 41467_2023_43726_MOESM1_ESM.pdf]

Prostate cancer genetic risk and associated aggressive disease in men of African ancestry

Pamela X.Y. Soh, Naledi Mmekwa, Desiree C. Petersen, Kazzem Gheybi, Smit van Zyl, Jue Jiang, Sean M. Patrick, Raymond Campbell, Weerachai Jaratlerdseri, Shingai B.A. Mutambirwa, M.S. Riana Bornman, Vanessa M. Hayes

Supplementary Information

Supplementary Data

Supplementary Data 1: Summary of clinical information for 113 South African men with prostate cancer where deep sequence data was available[1].

Supplementary Data 2: Risk allele frequency (RAF) in our South African population (sequence data and exome array data where available) compared to previously reported RAF in African ancestry controls (N=61,620)[2].

Supplementary Data 3: Risk allele frequency (RAF) in our South African population (N=113) for the top 136 associated variants from the Uganda prostate cancer GWAS study (UGPCS)[3] and for the African Ancestry prostate cancer study (AAPC) which were reported by Du et al., 2018[3]. None of these variants were genotyped in the exome array.

Supplementary Data 4: Risk allele frequencies (RAF) for the top 30 associated variants from the Ghana GWAS study[4] in South African PCa sequenced cases, and for the samples genotyped on the exomic array, where available.

Supplementary Data 5: Summary of clinical information for the exome study population. PSA, prostate-specific antigen; ISUP, International Society of Urological Pathology.

Supplementary Data 6: Risk allele frequencies, odds ratios (OR), and P-values for 397 known cancer variants out out of 2477 previously summarised[5] that were available on the exomic array.

Supplementary Data 7: Genes significantly associated to PCa in the rare variant gene-based analysis, and the frequencies of each set of genotypes in cases compared to controls, as well as predicted consequences of each variant.

Supplementary Data 8: Genes significantly associated to PCa in the gene-based analysis including common and rare variants, and the frequencies of each set of genotypes in cases compared to controls, as well as predicted consequences of each variant.

Supplementary Data 9: Genes significantly associated to HRPCa in the gene-based analysis including common and rare variants, and the frequencies of each set of genotypes in cases compared to controls, as well as predicted consequences of each variant.

Supplementary Data 10: Number of SNPs and proportion (out of 247,780 assayed variants) per minor allele frequency (MAF) interval for 780 samples genotyped on the Illumina HumanExome BeadChip v1.0 array, prior to and post-processing of rare variants (MAF≤0.01) with zCall v3.4[6].

Supplementary Figures

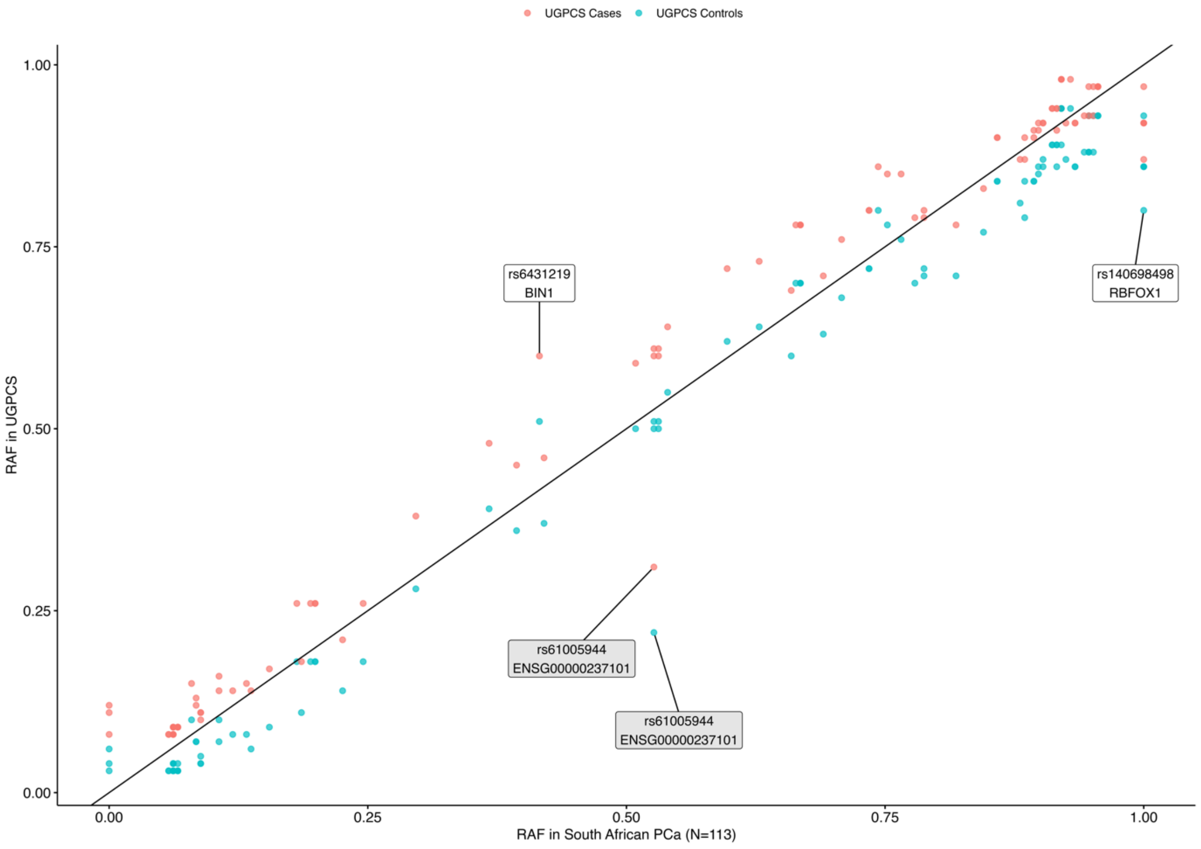

Supplementary Figure 1. Comparison of risk allele frequencies (RAF) for the top 136 associated variants for prostate cancer cases (N=571, red) and controls (N=485, blue) from the Uganda GWAS study[3] against our South African sequenced cases (N=113). The variants with the largest difference in RAF (>0.15) are labelled. Gene labels in white boxes indicate variants that overlap a gene, while gene labels in grey indicate the closest genes to the variant.

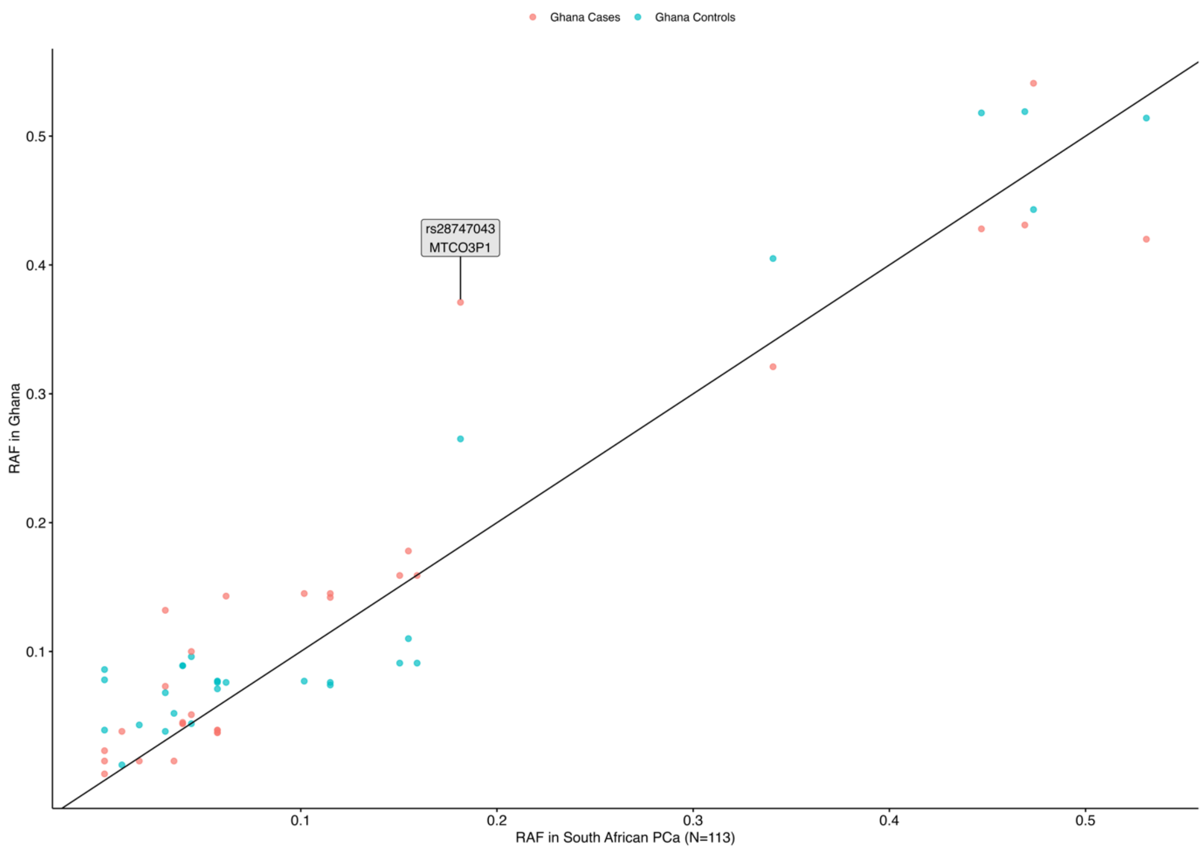

Supplementary Figure 2. Comparison of risk allele frequencies (RAF) for the top 30 associated variants for prostate cancer cases (N=474, red) and controls (N=458, blue) from the Ghana GWAS study[4] against our South African sequenced cases (N=113). One variant (labelled) had a difference in RAF >0.15. The grey box indicates the closest gene to the variant.

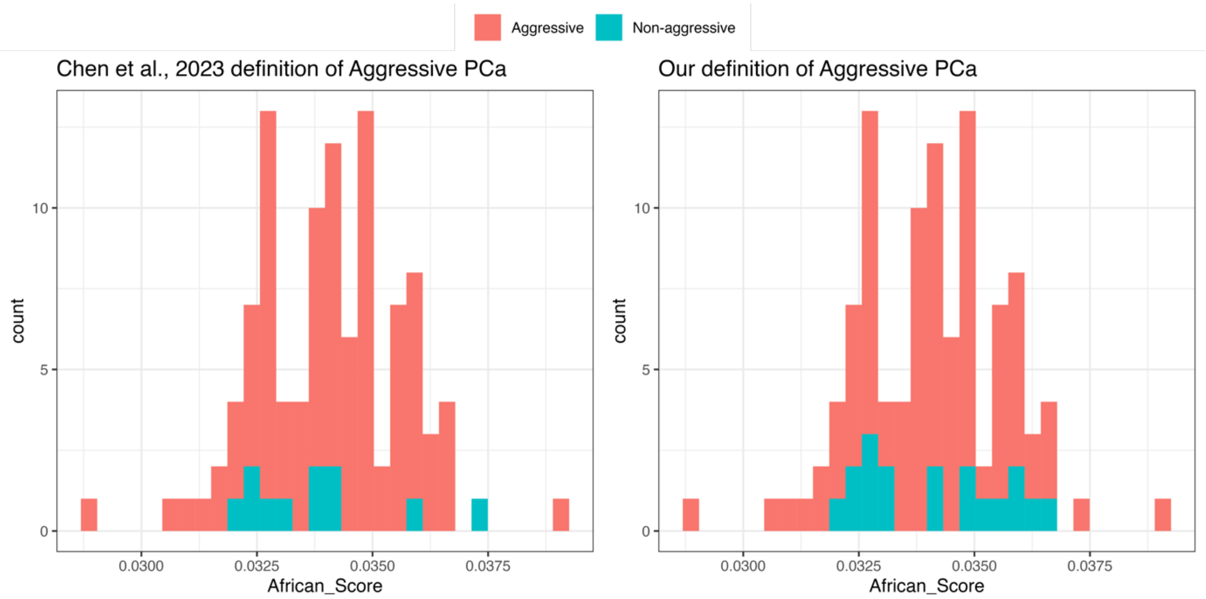

Supplementary Figure 3. Histogram of scores of the South African PCa cases using African weights[2], according to Chen et al., 2023's definition of aggressive PCa (ISUP 4-5 or PSA ≥ 20ng/ml; aggressive N=101, non-aggressive N=11) or our definition of aggressive PCa (ISUP 3-5; aggressive N=87, non-aggressive N=18).

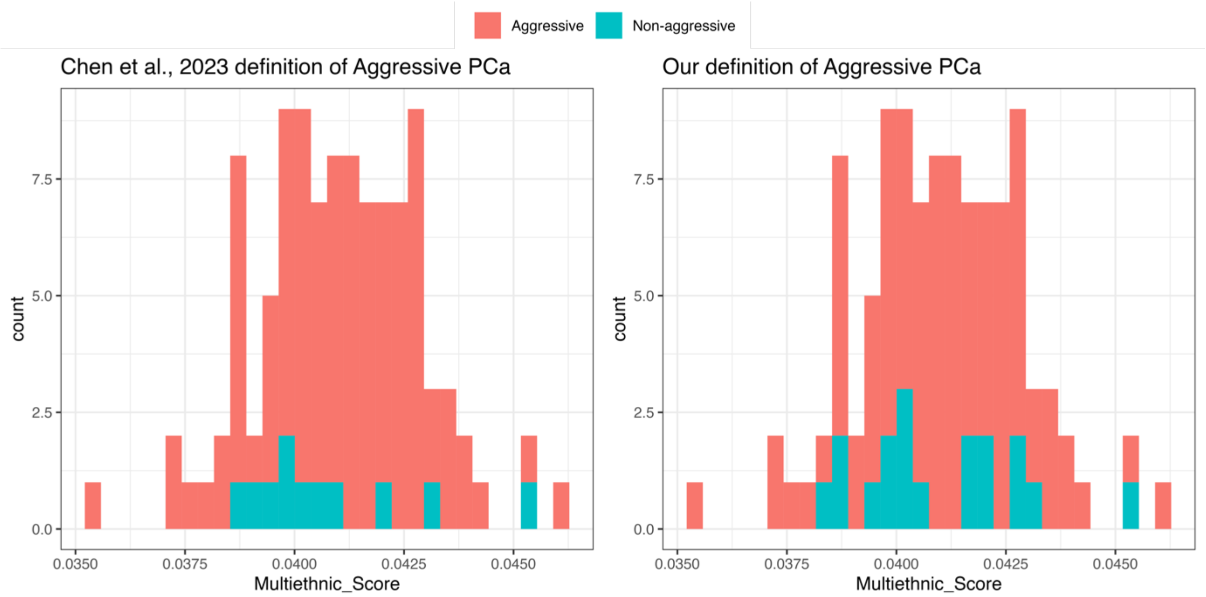

Supplementary Figure 4. Histogram of scores of the South African PCa cases using multiethnic weights[2], according to Chen et al., 2023's definition of aggressive PCa (ISUP 4-5 or PSA ≥ 20ng/ml; aggressive N=101, non-aggressive N=11) or our definition of aggressive PCa (ISUP 3-5; aggressive N=87, non-aggressive N=18).

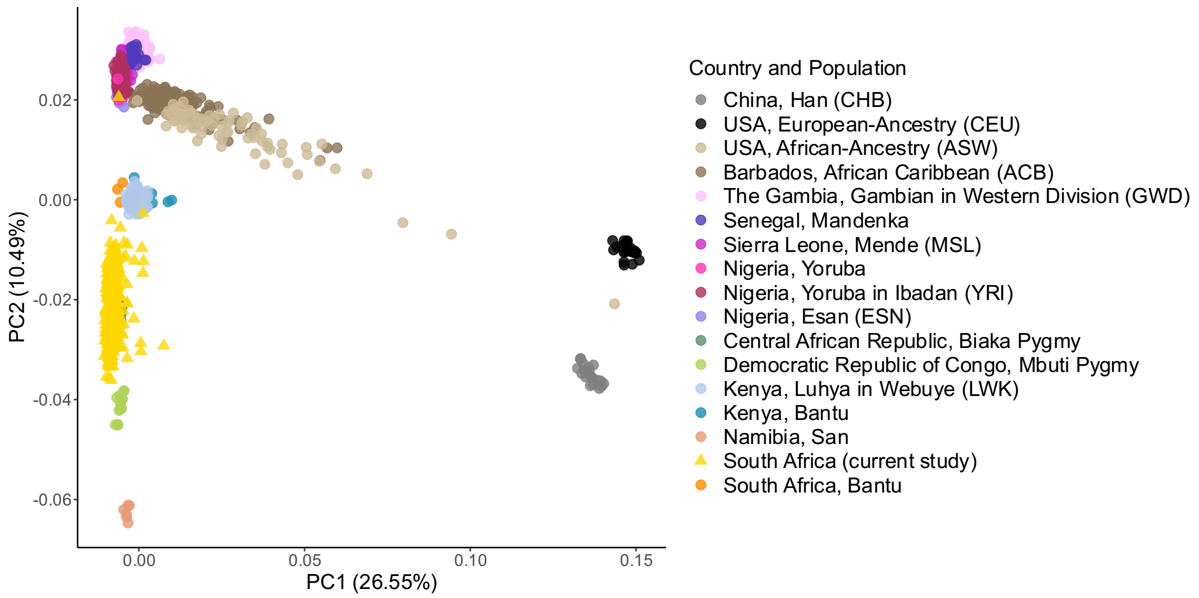

Supplementary Figure 5. Principal Component Analysis (PCA) plot of the the Human Diversity Genome Project (HDGP) and 1000 Genomes Project (1KGP) subset of gnomAD v3.1.2 individuals (circles) of African-ancestry (N=1003), European-ancestry from USA (CEU; N=20) and Han Chinese (CHB, N=20)[7], with the samples from the current study (yellow triangles, N=781).

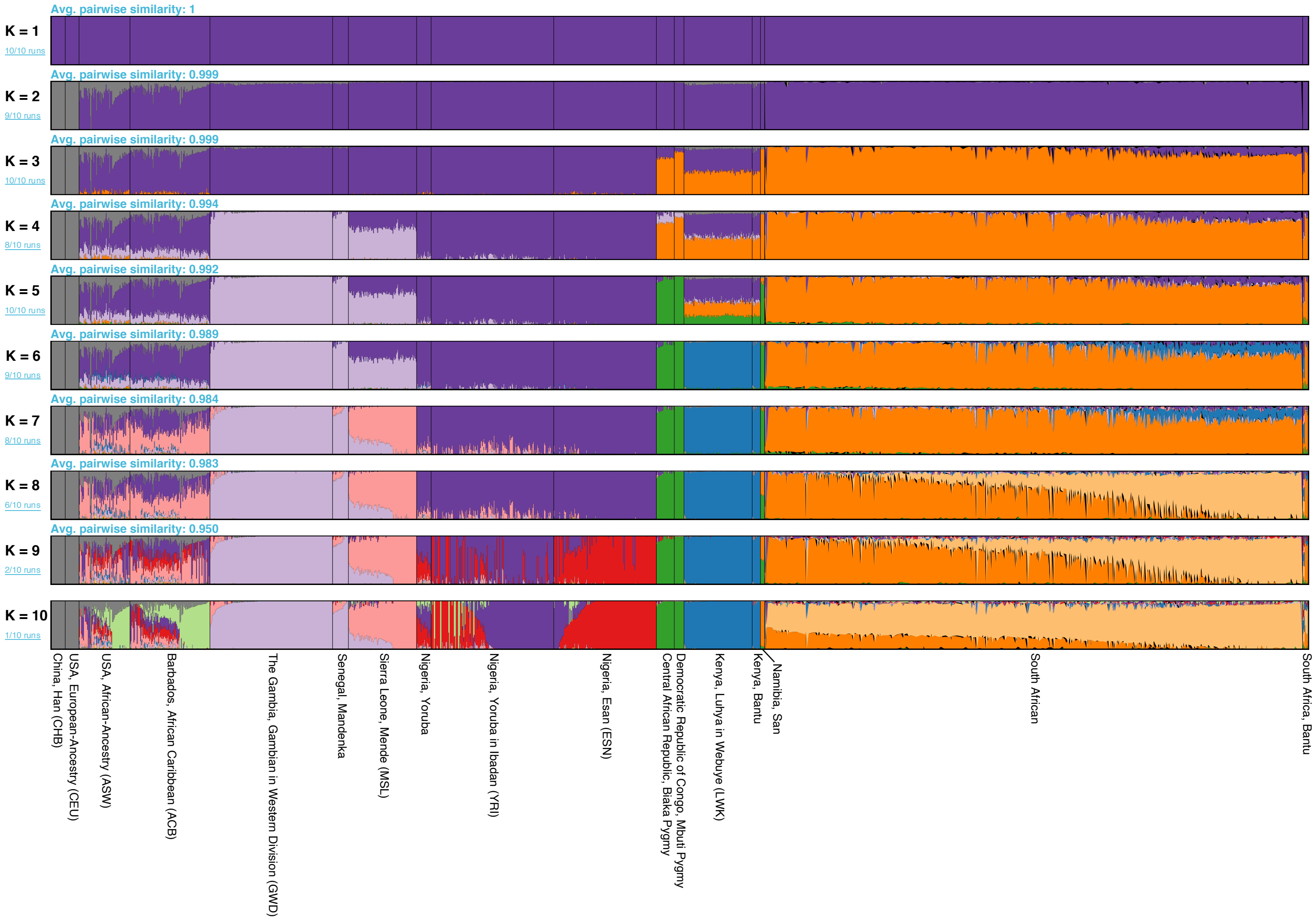

Supplementary Figure 6. ADMIXTURE plots for K=1 to K=10 based on the linkage-pruned exome array SNPs (N=77,372). The number of runs replicated by the plot per K value is indicated on the left, with average pairwise similarity between the runs indicated above each plot. Samples from the current study are labelled as "South African", while all other samples were extracted from the Human Diversity Genome Project (HDGP) and 1000 Genomes Project (1KGP) subset of gnomAD v3.1.2[7].

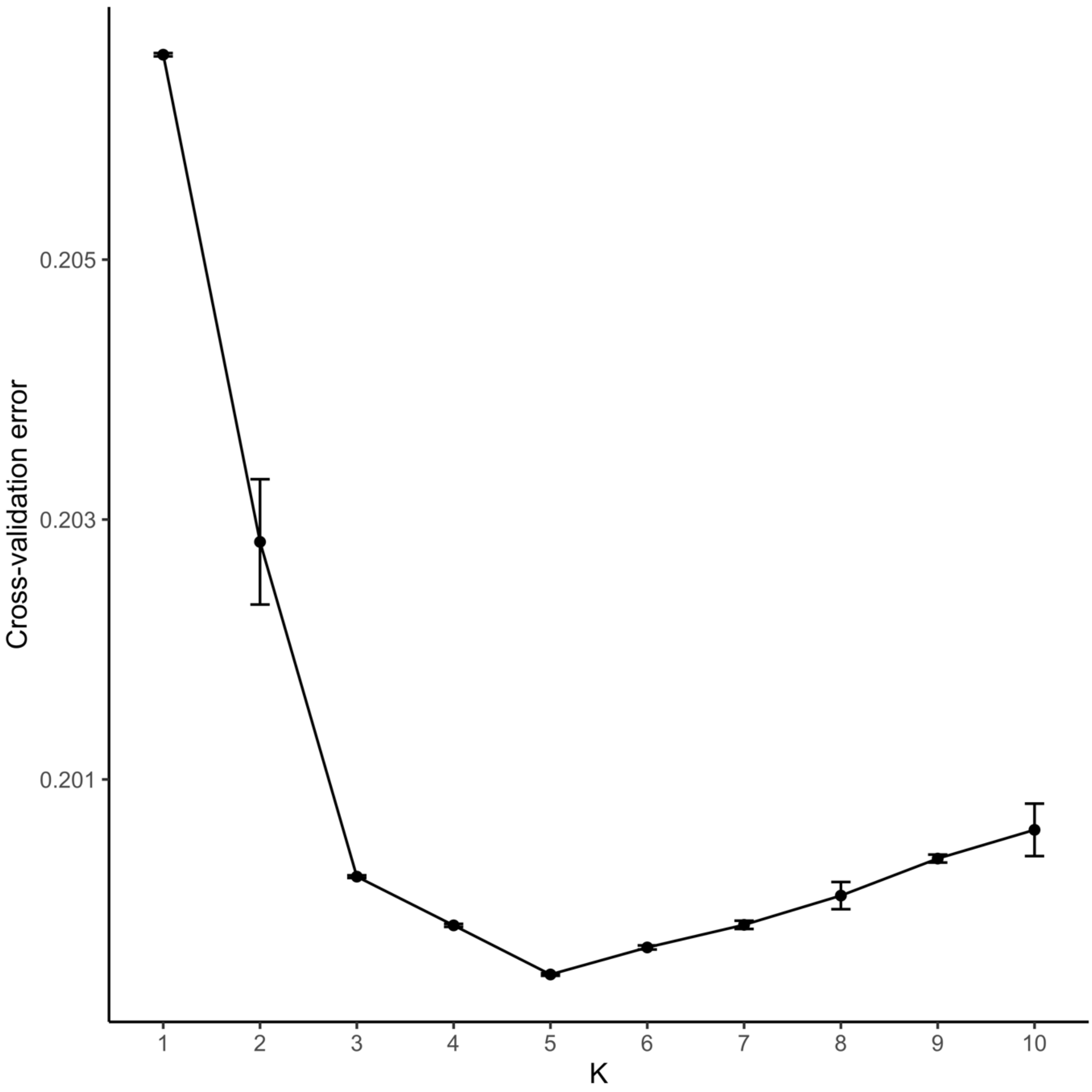

Supplementary Figure 7. Cross-validation error rates for ADMIXTURE runs (K=1 to K=10). Circles indicate mean values and error bars indicate standard deviation from 10 runs per value of K. K=5 produced the lowest cross-validation error at 0.16182.

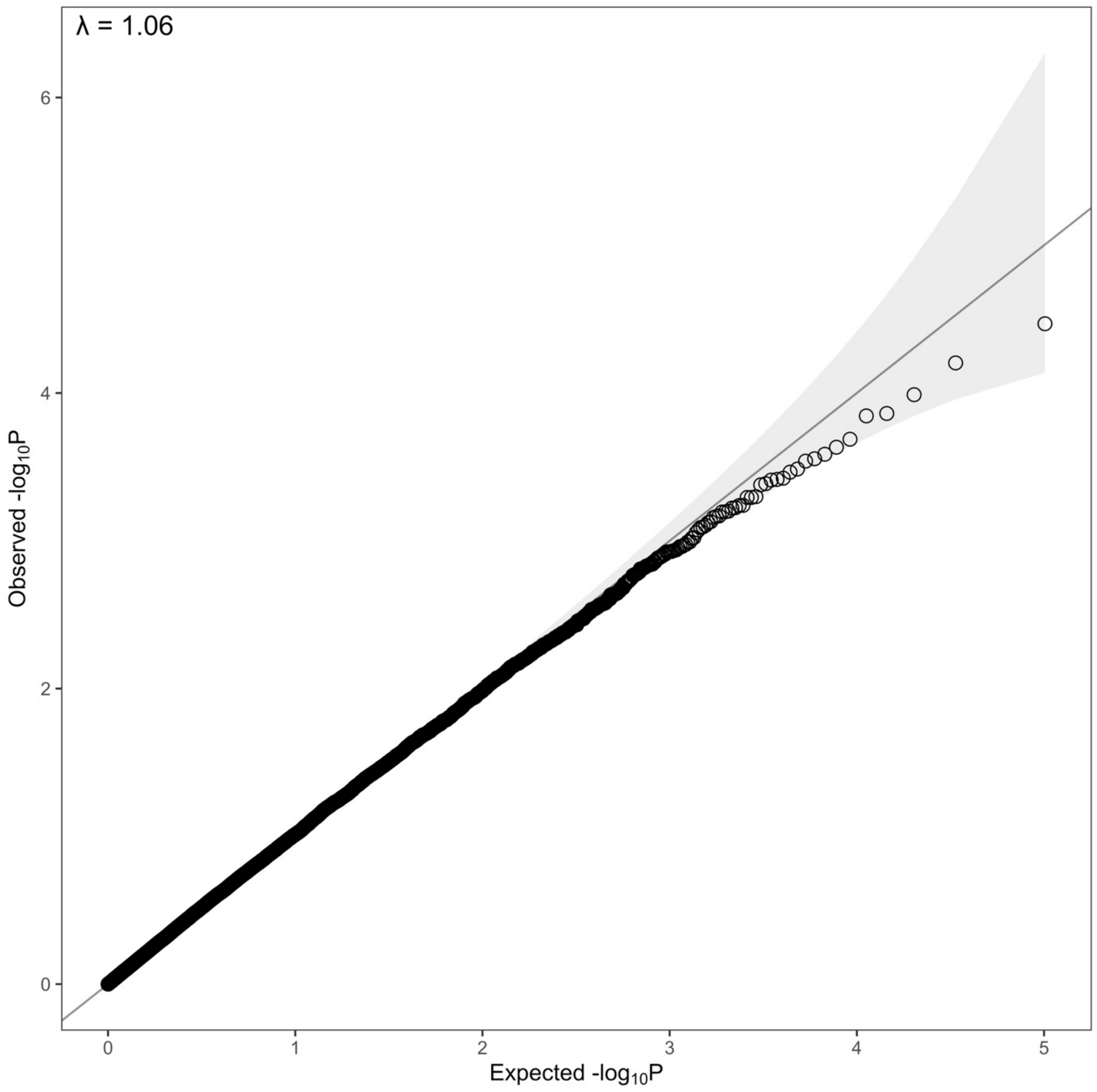

Supplementary Figure 8. Quantile-quantile plot of observed and expected -$\log_{10}$ P values for the age-adjusted regression analysis with all cases (N=451) and controls (N=292). The grey band indicates the 95% confidence interval. The genomic inflation factor (λ) was 1.06.

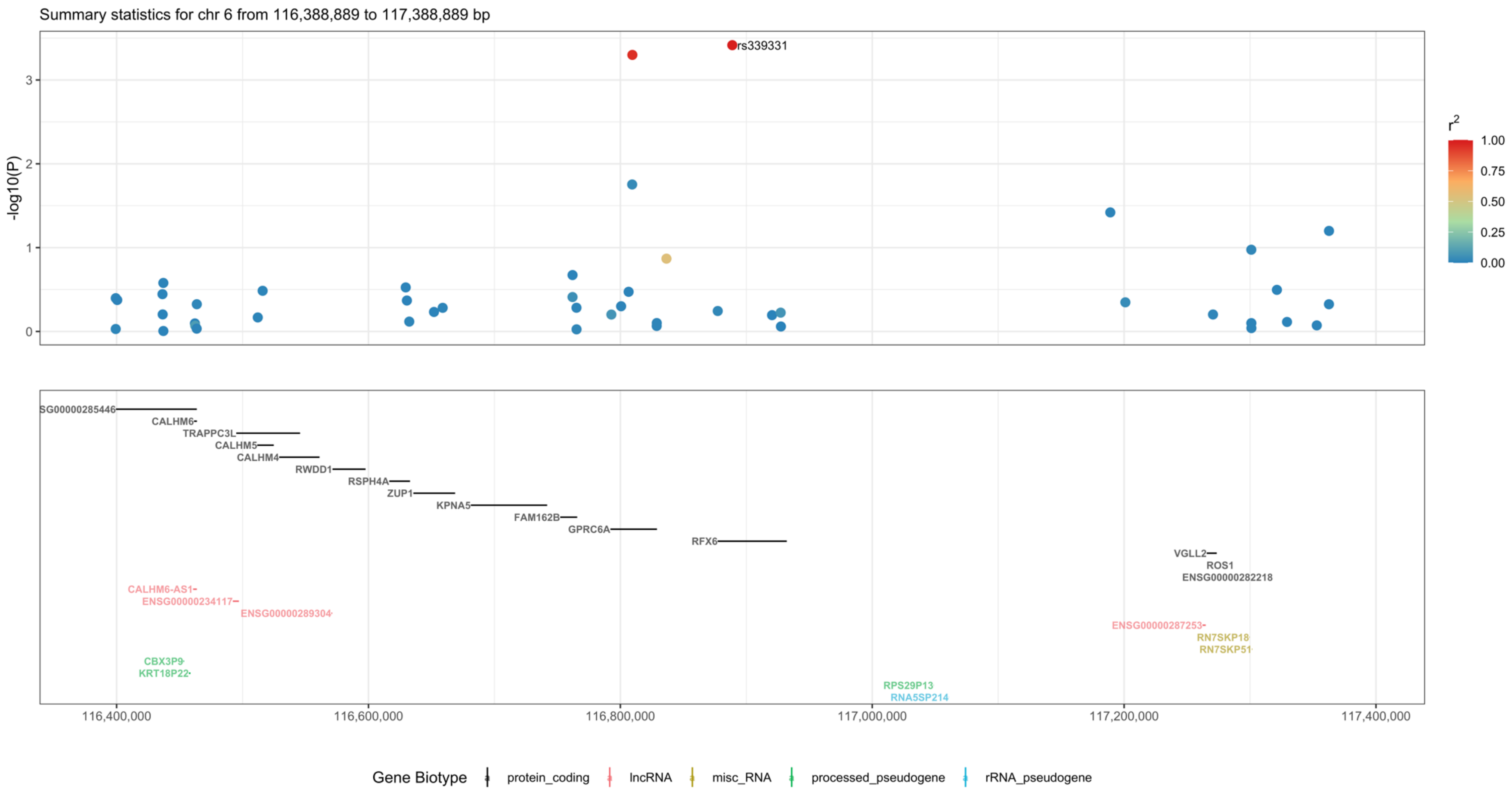

Supplementary Figure 9. Regional association plot showing linkage between rs339331 in *RFX6* to SNPs in the region. The top panel shows -$\log_{10}$ P-values from the GWAS comparing all cases (N=451) against controls (N=292), colour coded by linkage (r^2) to the top associated SNP in the region (labelled). The bottom panel shows the genes in the region, colour coded by gene biotype (fetched from Ensembl Human GRCh38.p13, version 108). Variants in strong to moderate linkage to rs339331 include rs2274911 (r^2=0.95) and rs636252 (r^2=0.54).

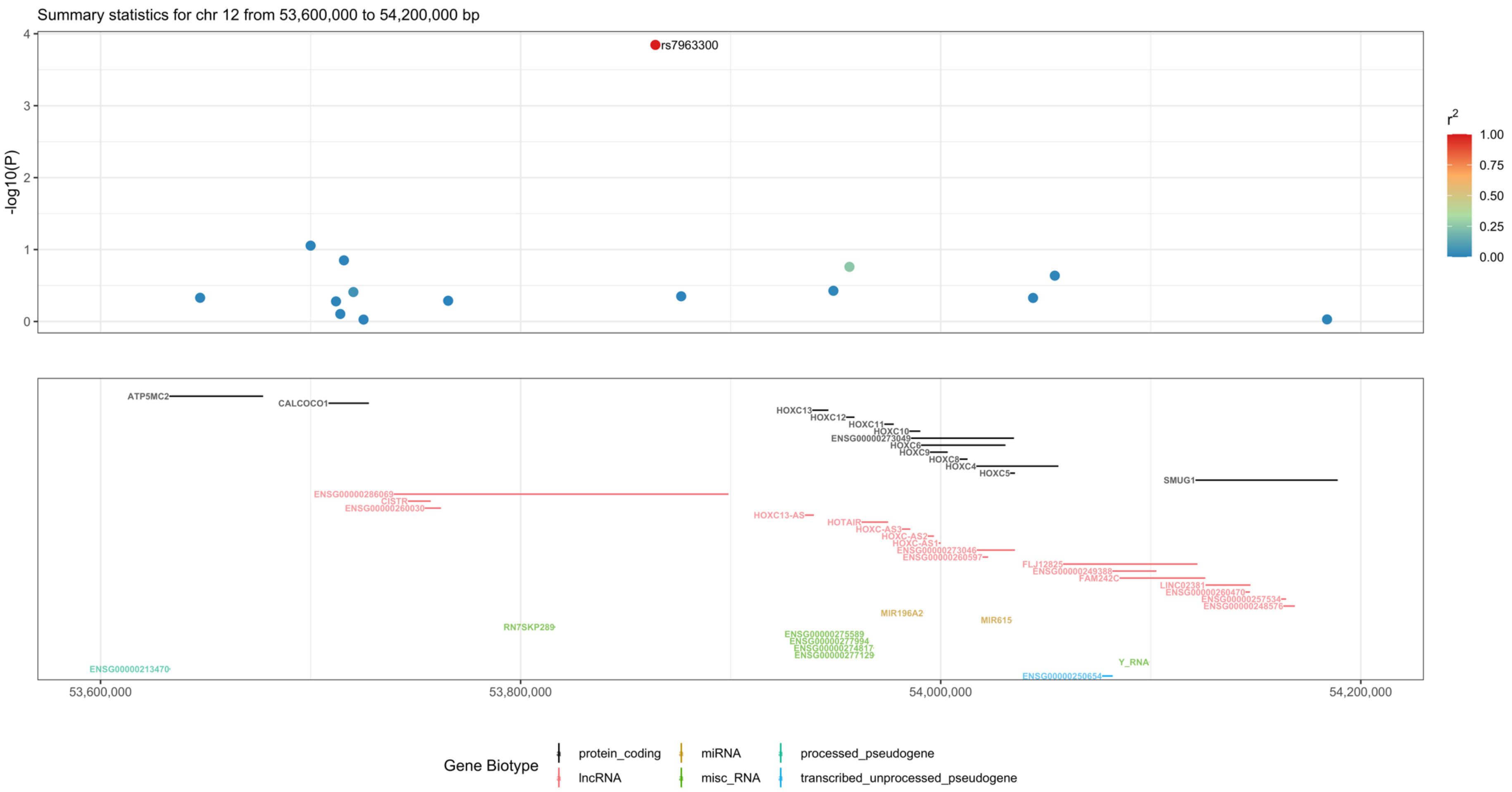

Supplementary Figure 10. Regional association plot showing linkage of rs7963300 to nearby SNPs and proximity to *HOXC* genes. The top panel shows -log$_{10}$ P-values from the GWAS comparing all cases (N=451) against controls (N=292), colour coded by linkage (r^2) to the top associated SNP in the region (labelled). The bottom panel shows the genes in the region, colour coded by gene biotype (fetched from Ensembl Human GRCh38.p13, version 108).

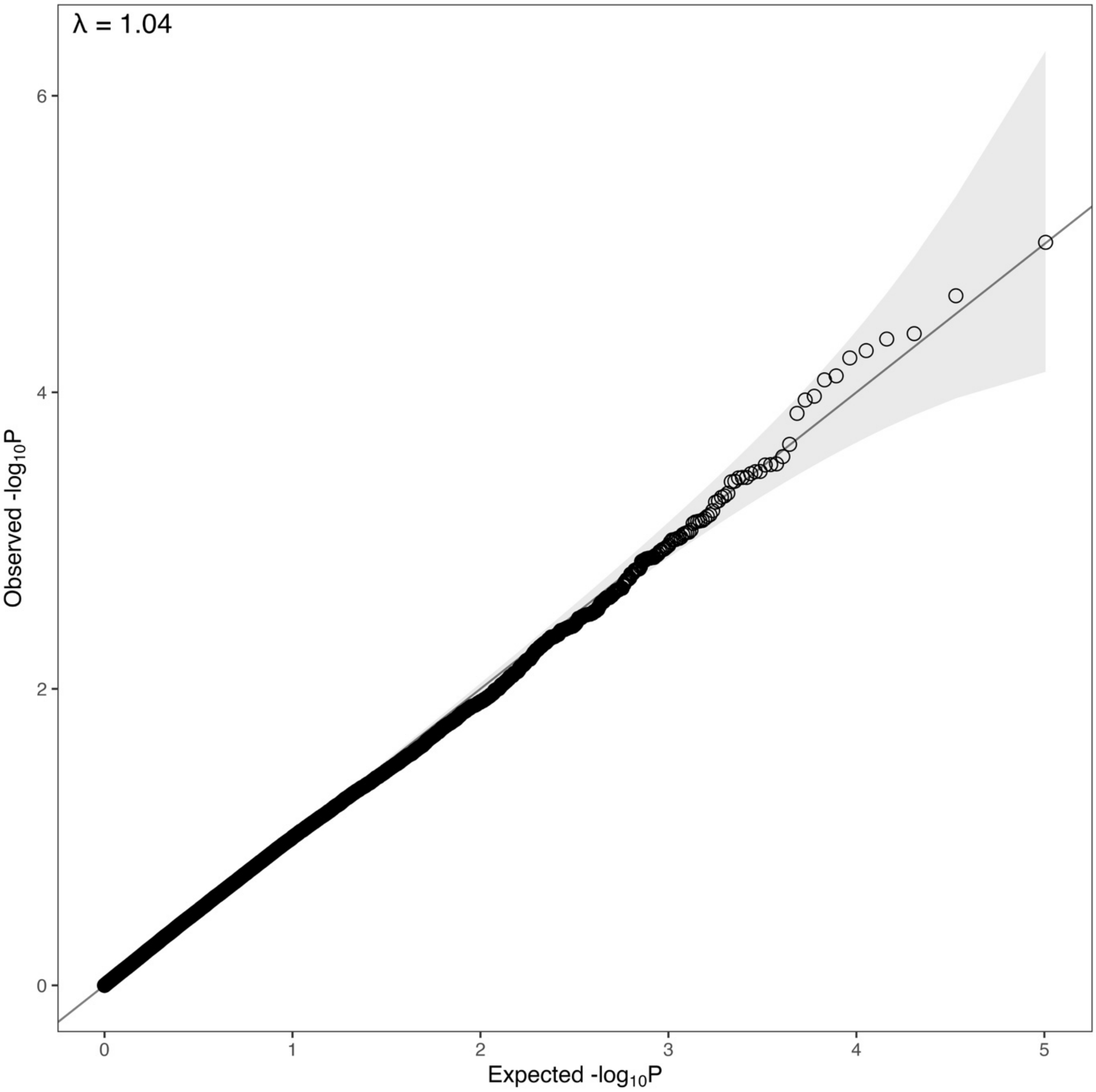

Supplementary Figure 11. Quantile-quantile plot of observed and expected $-\log_{10}$ P values for the age-adjusted regression analysis with high risk PCa (ISUP 3-5) (N=203) against low risk or no PCa (N=461). The grey band indicates the 95% confidence interval. The genomic inflation factor (λ) was 1.04.

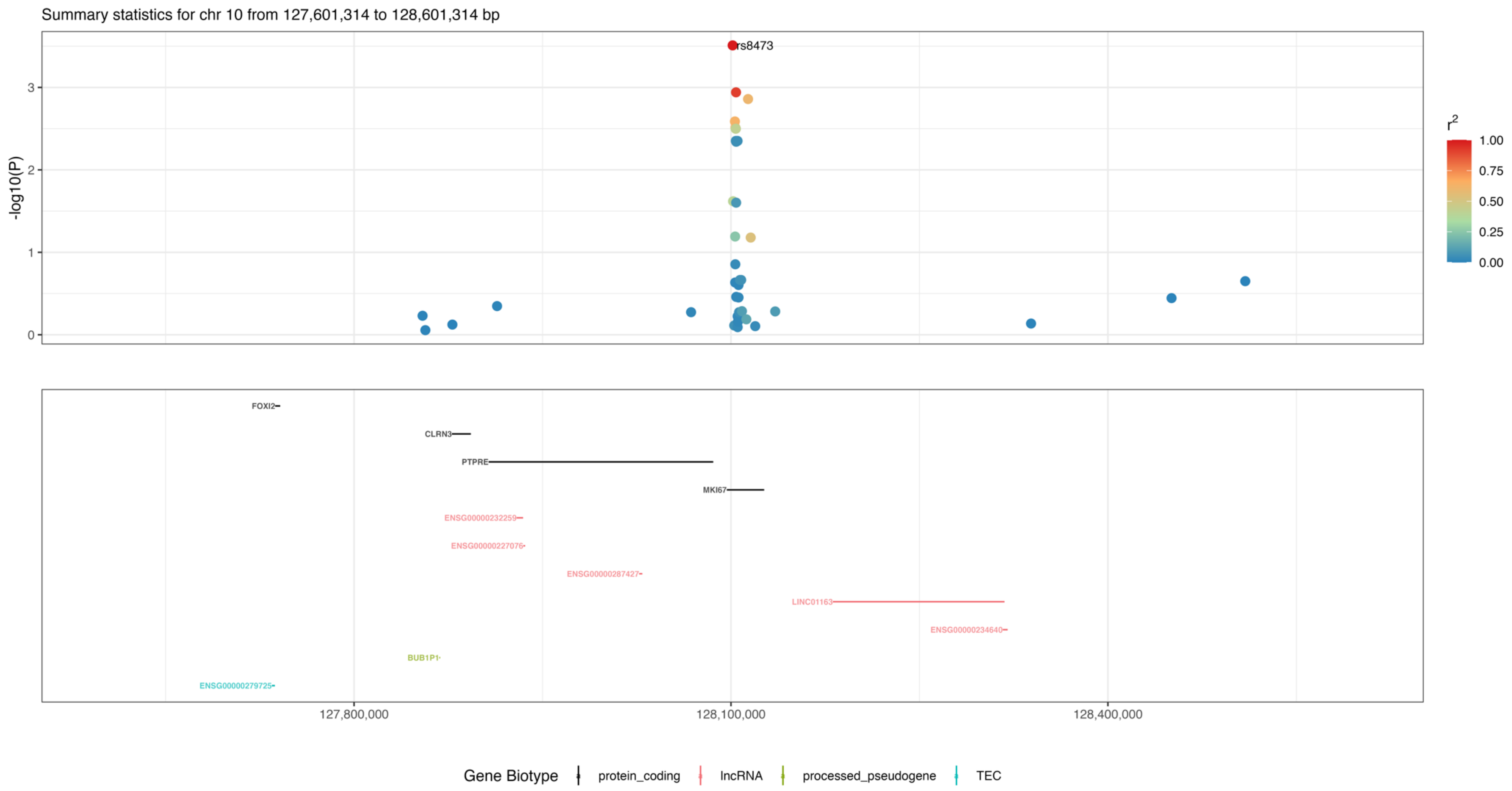

Supplementary Figure 12. Regional association plot showing linkage of rs8473 to nearby SNPs. The top panel shows $-\log_{10}$ P-values from the GWAS comparing high-risk PCa cases (ISUP 3-5; N=203) against low-risk or no PCa (N=461), colour coded by linkage (r^2) to the top associated SNP in the region (labelled). The bottom panel shows the genes in the region, colour coded by gene biotype (fetched from Ensembl Human GRCh38.p13, version 108). The variant in strong linkage to rs8473 is rs1063535 (r^2=0.92), while variants in moderate linkage include rs34750407, rs11016071, rs10082391, rs1050767, rs12777740, rs11016076, and rs7095325 (r^2=0.4 to 0.64).

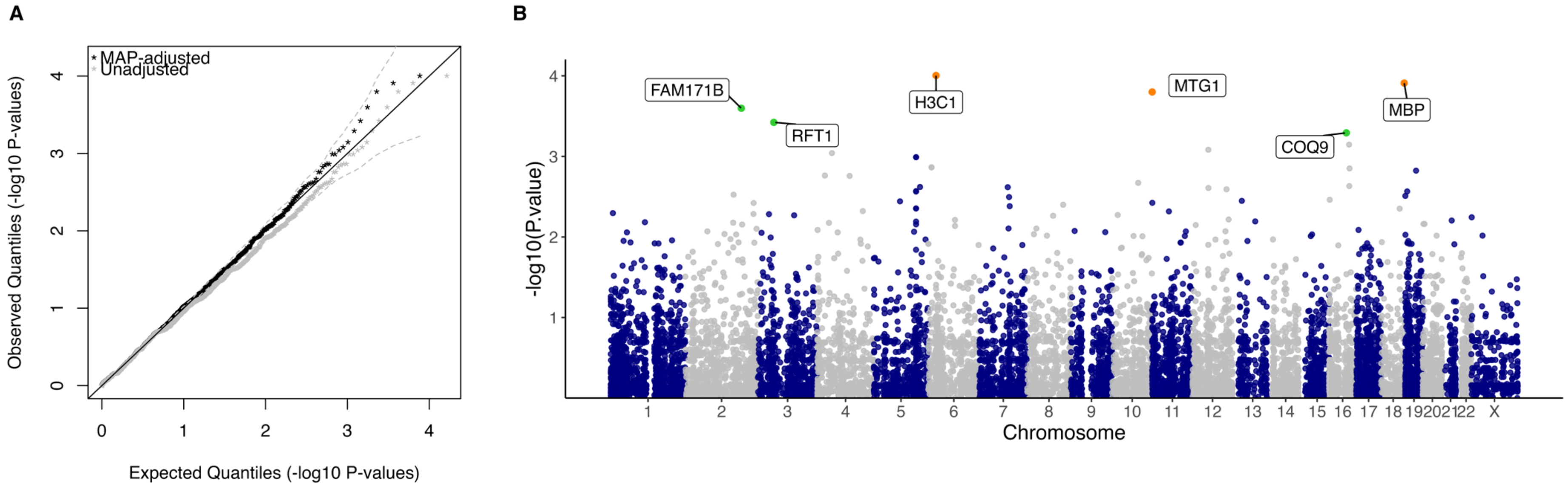

C

Gene	P value	N Marker All	N Marker Test	MAC	m	Method bin	MAP
H3C1	9.91e-05	1	1	9	9	ER	9.91e-05
MBP	1.23e-04	1	1	12	12	ER	5.76e-06
MTG1	1.59e-04	3	3	17	17	ER	4.52e-08
FAM171B	2.52e-04	2	2	8	8	ER	2.52e-04
RFT1	3.78e-04	3	3	17	17	ER	5.12e-08
COQ9	5.10e-04	3	3	14	10	ER	3.88e-05

Supplementary Figure 13. Results of gene-based rare variant analysis using the optimal unified sequence kernel association test (SKAT-O) for PCa (451 cases, 292 controls; 31,345 SNPs in 11,740 genes). (A) Quantile-quantile plot showing unadjusted and minimum achievable P-value (MAP) adjusted values. (B) Manhattan plot of P-values from the analysis, with the top associated genes labelled. Each dot represents a gene, with significant genes (family-wise error rate cut-off = 0.05, calculated per chromosome) indicated in orange, and other top associated genes in green. (C) Table of the top associated genes in the analysis, including P values, minor allele count (MAC), number of individuals carrying the minor allele (m), and MAP. ER in the method bin indicates the efficient resampling (ER) method was used to compute p-values.

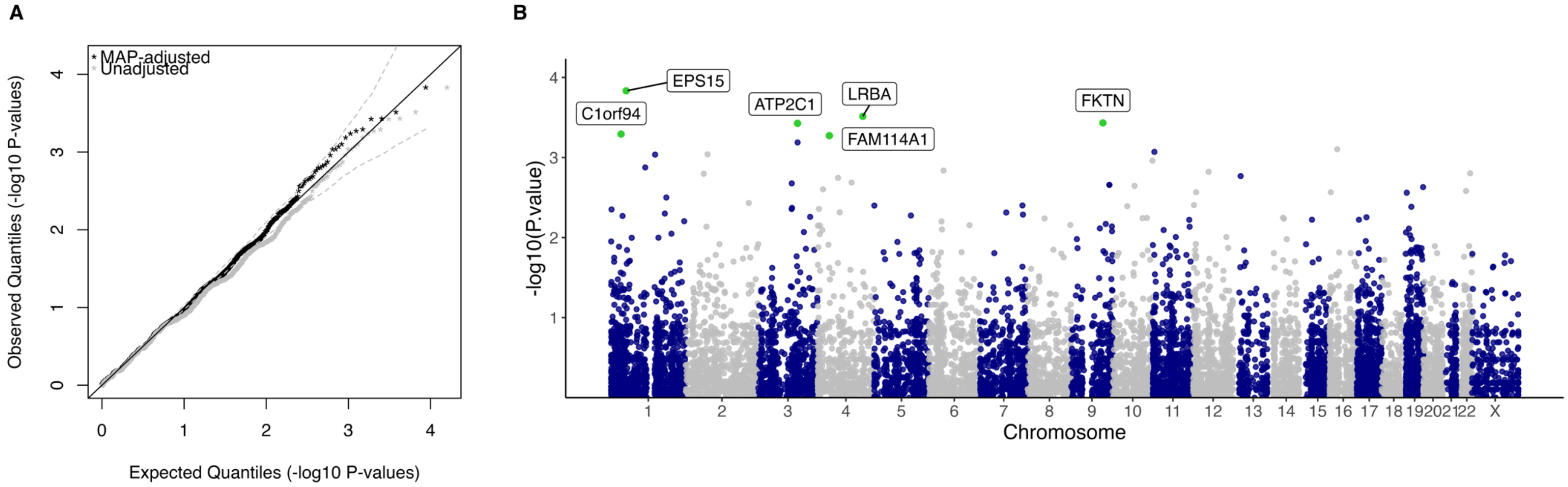

C

Gene	P value	N Marker All	N Marker Test	MAC	m	Method bin	MAP
EPS15	1.47e-04	3	3	17	17	ER	4.15e-10
LRBA	3.07e-04	15	14	50	43	QA	-1.00e+00
FKTN	3.70e-04	2	2	6	6	ER	3.70e-04
ATP2C1	3.75e-04	1	1	6	6	ER	3.75e-04
C1orf94	5.10e-04	2	2	7	7	ER	1.18e-04
FAM114A1	5.34e-04	3	3	17	17	ER	5.41e-10

Supplementary Figure 14. Results of gene-based rare variant analysis using the SKAT-O test for HRPCa (203 HRPCa, 461 LRPCa/no PCa; 31,345 SNPs in 11,740 genes). (A) Quantile-quantile plot showing unadjusted and minimum achievable P-value (MAP) adjusted values. (B) Manhattan plot of P-values from the analysis, with the top associated genes labelled. Each dot represents a gene, with the top associated genes in green. (C) Table of the top associated genes in the analysis, including P values, minor allele count (MAC), number of individuals carrying the minor allele (m), and MAP. ER in the method bin indicates the efficient resampling (ER) method was used to compute p-values, while QA indicates quantile adjusted moment matching. Note no genes were significant (family-wise error rate cut-off = 0.05, calculated per chromosome) in this analysis.

A

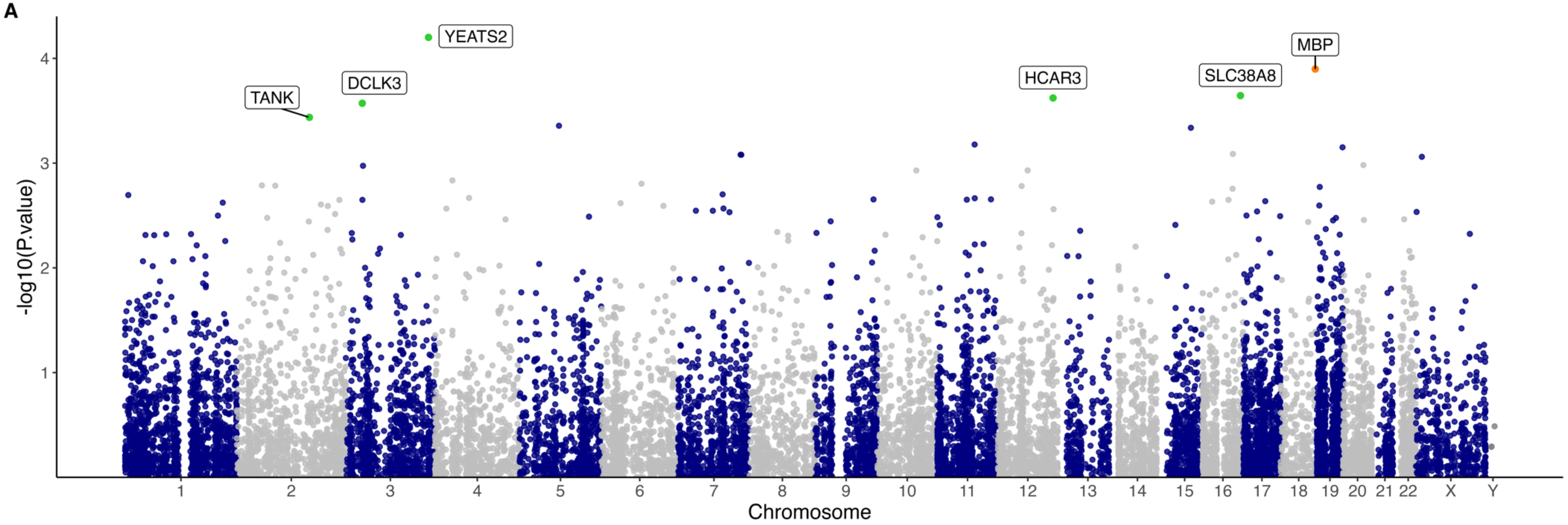

B

Gene	P value	Q	N Marker All	N Marker Test	N Marker Rare	N Marker Common
YEATS2	6.30e-05	7.52	8	8	3	5
MBP	1.26e-04	7.98	6	6	1	5
SLC38A8	2.27e-04	8.27	10	10	8	2
HCAR3	2.39e-04	616.48	1	1	0	1
DCLK3	2.68e-04	7.49	6	6	5	1
TANK	3.66e-04	7.17	4	4	1	3

Supplementary Figure 15. Results of gene-based analysis using the SKAT-O test including common and rare variants for PCa (451 cases, 292 controls; 71,908 SNPs in 15,593 genes). (A) Manhattan plot of P-values from the analysis, with the top associated genes labelled. Each dot represents a gene, with significant genes (family-wise error rate cut-off = 0.05, calculated per chromosome) indicated in orange, and other top associated genes in green. (B) Table of the top associated genes in the analysis including P values, Q values, and the number of rare and common markers included.

A

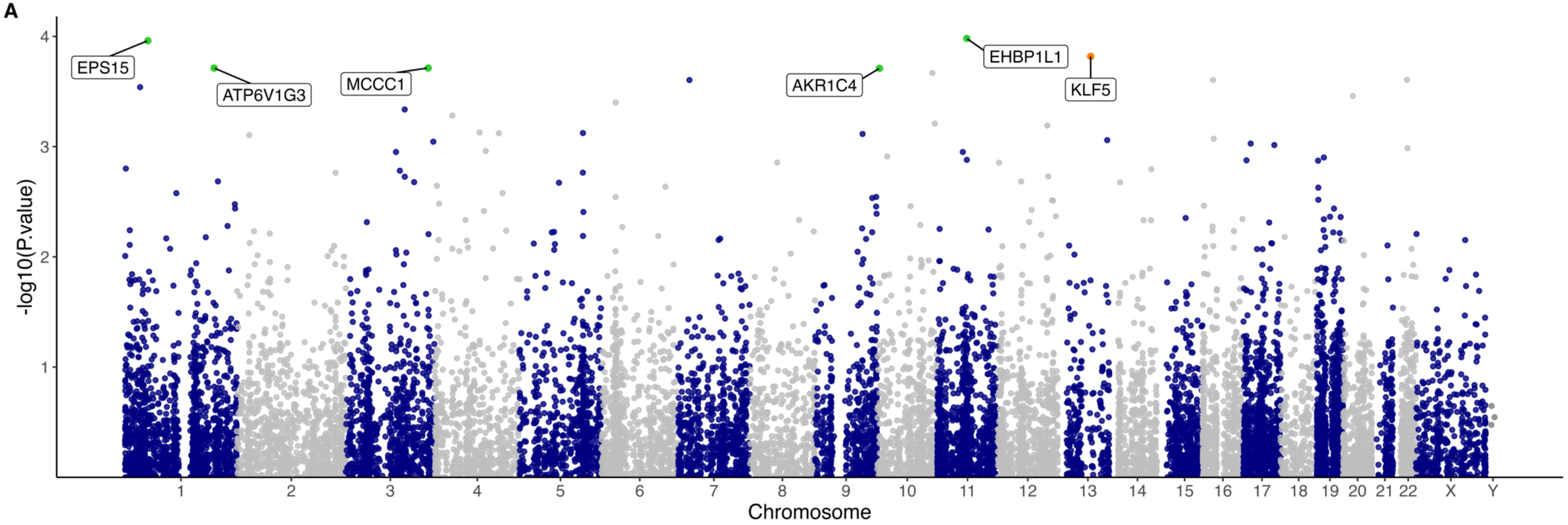

B

Gene	P value	Q	N Marker All	N Marker Test	N Marker Rare	N Marker Common
EHBP1L1	1.04e-04	8.93	7	7	4	3
EPS15	1.09e-04	9.35	4	4	3	1
KLF5	1.52e-04	6.95	4	4	1	3
MCCC1	1.94e-04	6.66	6	6	2	4
ATP6V1G3	1.94e-04	6.96	5	5	2	3
AKR1C4	1.95e-04	112.68	5	5	0	5

Supplementary Figure 16. Results of gene-based analysis using the SKAT-O test including common and rare variants for HRPCa (203 HRPCa, 461 LRPCa/no PCa; 71,908 SNPs in 15,593 genes). (A) Manhattan plot of P-values from the analysis, with the top associated genes labelled. Each dot represents a gene, with significant genes (family-wise error rate cut-off = 0.05, calculated per chromosome) indicated in orange, and other top associated genes in green. (B) Table of the top associated genes in the analysis including P values, Q values, and the number of rare and common markers included.

Supplementary References

1. Jaratlerdsiri W*, et al.* African-specific molecular taxonomy of prostate cancer. *Nature* **609**, 552-559 (2022).

2. Chen F*, et al.* Evidence of Novel Susceptibility Variants for Prostate Cancer and a Multiancestry Polygenic Risk Score Associated with Aggressive Disease in Men of African Ancestry. *Eur Urol* **84**, 13-21 (2023).

3. Du Z*, et al.* Genetic risk of prostate cancer in Ugandan men. *Prostate* **78**, 370-376 (2018).

4. Cook MB*, et al.* A genome-wide association study of prostate cancer in West African men. *Hum Genet* **133**, 509-521 (2014).

5. Harlemon M*, et al.* A Custom Genotyping Array Reveals Population-Level Heterogeneity for the Genetic Risks of Prostate Cancer and Other Cancers in Africa. *Cancer Res* **80**, 2956-2966 (2020).

6. Goldstein JI*, et al.* zCall: a rare variant caller for array-based genotyping: genetics and population analysis. *Bioinformatics (Oxford, England)* **28**, 2543-2545 (2012).

7. Karczewski KJ*, et al.* The mutational constraint spectrum quantified from variation in 141,456 humans. *Nature* **581**, 434-443 (2020).
