# Supplementary material for: Prostate cancer genetic risk and associated aggressive disease in men of African ancestry
Source: Nat Commun. 2023 Dec 5;14:8037. doi: 10.1038/s41467-023-43726-w (PMC10697980; doi:10.1038/s41467-023-43726-w)
Supplement: Supplementary file 3 — Description of Additional Supplementary Files [file 41467_2023_43726_MOESM3_ESM.pdf]

Description of Additional Supplementary Files

File Name: Supplementary Data 1
Description: Summary of clinical information for 113 South African men with prostate cancer where deep sequence data was available (Jaratlerdsiri et al., 2022).

File Name: Supplementary Data 2
Description: Risk allele frequency (RAF) in our South African population (sequence data and exome array data where available) compared to previously reported RAF in African ancestry controls (N=61,620) (Chen et al., 2023).

File Name: Supplementary Data 3
Description: Risk allele frequency (RAF) in our South African population (N=113) for the top 136 associated variants from the Uganda prostate cancer GWAS study (UGPCS) (Du et al., 2018) and for the African Ancestry prostate cancer study (AAPC) which were reported by Du et al., 2018. None of these variants were genotyped in the exome array.

File Name: Supplementary Data 4
Description: Risk allele frequencies (RAF) for the top 30 associated variants from the Ghana GWAS study (Cook et al., 2014) in South African PCa sequenced cases, and for the samples genotyped on the exomic array, where available.

File Name: Supplementary Data 5
Description: Summary of clinical information for the exome study population. PSA, prostate-specific antigen; ISUP, International Society of Urological Pathology

File Name: Supplementary Data 6
Description: Risk allele frequencies, odds ratios (OR), and P-values for 397 known cancer variants out out of 2477 previously summarised (Harlemon et al., 2020) that were available on the exomic array.

File Name: Supplementary Data 7
Description: Genes significantly associated to PCa in the rare variant gene-based analysis, and the frequencies of each set of genotypes in cases compared to controls, as well as predicted consequences of each variant.

File Name: Supplementary Data 8
Description: Genes significantly associated to PCa in the gene-based analysis including common and rare variants, and the frequencies of each set of genotypes in cases compared to controls, as well as predicted consequences of each variant.

File Name: Supplementary Data 9
Description: Genes significantly associated to HRPCa in the gene-based analysis including common and rare variants, and the frequencies of each set of genotypes in cases compared to controls, as well as predicted consequences of each variant.

File Name: Supplementary Data 10

Description: Number of SNPs and proportion (out of 247,780 assayed variants) per minor allele frequency (MAF) interval for 780 samples genotyped on the Illumina HumanExome BeadChip v1.0 array, prior to and post-processing of rare variants (MAF£0.01) with zCall v3.4 (Goldstein et al., 2012).
