# Supplementary material for: Prostate cancer genetic risk and associated aggressive disease in men of African ancestry
Source: Nat Commun. 2023 Dec 5;14:8037. doi: 10.1038/s41467-023-43726-w (PMC10697980; doi:10.1038/s41467-023-43726-w)
Supplement: Supplementary file 14 — Reporting Summary [file 41467_2023_43726_MOESM14_ESM.pdf]

nature portfolio

Corresponding author(s): Vanessa M. Hayes

Last updated by author(s): 17 November 2023

Reporting Summary

Nature Portfolio wishes to improve the reproducibility of the work that we publish. This form provides structure for consistency and transparency in reporting. For further information on Nature Portfolio policies, see our Editorial Policies and the Editorial Policy Checklist.

Statistics

For all statistical analyses, confirm that the following items are present in the figure legend, table legend, main text, or Methods section.

n/a	Confirmed	
☐	☒	The exact sample size (*n*) for each experimental group/condition, given as a discrete number and unit of measurement
☐	☒	A statement on whether measurements were taken from distinct samples or whether the same sample was measured repeatedly
☐	☒	The statistical test(s) used AND whether they are one- or two-sided<br>*Only common tests should be described solely by name; describe more complex techniques in the Methods section.*
☐	☒	A description of all covariates tested
☐	☒	A description of any assumptions or corrections, such as tests of normality and adjustment for multiple comparisons
☐	☒	A full description of the statistical parameters including central tendency (e.g. means) or other basic estimates (e.g. regression coefficient) AND variation (e.g. standard deviation) or associated estimates of uncertainty (e.g. confidence intervals)
☐	☒	For null hypothesis testing, the test statistic (e.g. *F*, *t*, *r*) with confidence intervals, effect sizes, degrees of freedom and *P* value noted<br>*Give P values as exact values whenever suitable.*
☐	☒	For Bayesian analysis, information on the choice of priors and Markov chain Monte Carlo settings
☒	☐	For hierarchical and complex designs, identification of the appropriate level for tests and full reporting of outcomes
☐	☒	Estimates of effect sizes (e.g. Cohen's *d*, Pearson's *r*), indicating how they were calculated

Our web collection on statistics for biologists contains articles on many of the points above.

Software and code

Policy information about availability of computer code

Data collection	No software was used to collect the data
Data analysis	All software used was open source. Software used included Illumina GenomeStudio v2.0, PLINK v1.9, RStudio v4.1.1 (including packages ggplot2 v3.4.1, biomaRt v2.50.3, qvalue v2.26, SKAT v2.2.4), ADMIXTURE v1.3.0, pong v1.5, zCall v3.4, and GCTA v1.92.0.<br>Publicly available scripts from William Rayner from the Wellcome Centre for Human Genetics, Oxford website (https://www.well.ox.ac.uk/~wrayner/strand/), as well as the web tool UCSC Table Browser were utilised in the data analysis.

For manuscripts utilizing custom algorithms or software that are central to the research but not yet described in published literature, software must be made available to editors and reviewers. We strongly encourage code deposition in a community repository (e.g. GitHub). See the Nature Portfolio guidelines for submitting code & software for further information.

Data

Policy information about availability of data

All manuscripts must include a data availability statement. This statement should provide the following information, where applicable:

- Accession codes, unique identifiers, or web links for publicly available datasets
- A description of any restrictions on data availability
- For clinical datasets or third party data, please ensure that the statement adheres to our policy

The sequencing data analysed in this study were obtained from the European Genome-Phenome Archive (EGA; https://ega-archive.org/) under overarching

accession EGAS00001006425, with access to the Southern African Prostate Cancer Study (SAPCS) Dataset (EGAD00001009067) granted by the SAPCS Data Access Committee (DAC). Exomic genotyping summary statistics have been deposited in the GWAS Catalog database (www.ebi.ac.uk/gwas) under accession code GCST90296485 for cases versus control data and GCST90296486 for high-risk PCa versus low-risk PCa and control. Polygenic risk scores are available in the PGS Catalog database (https://www.pgscatalog.org/) under accession code PGP000516. Source data are provided with this paper.

Research involving human participants, their data, or biological material

Policy information about studies with human participants or human data. See also policy information about sex, gender (identity/presentation), and sexual orientation and race, ethnicity and racism.

Reporting on sex and gender	All samples used in this study were of the male sex.
Reporting on race, ethnicity, or other socially relevant groupings	Samples originating from this study (798 samples genotyped on the Illumina HumanExome Beadchip array) self-identified ethno-linguistically, by two generations, as Black South African. Only their country of origin (South Africa) was used to compare to external data (gnomAD v3.1.2 Human Genome Diversity Project (HGDP) and 1000 Genomes Project (1KGP) subset) for ethnicity differences.
Population characteristics	A total of 798 men were recruited from Southern African Prostate Cancer Study (SAPCS) presentative urology clinics within South Africa. Excluding samples removed during quality control (duplicates, missing age, admixed with other ethnicities), cases were defined as presenting with clinicopathologically confirmed PCa (N=451) and controls with no evidence of cancer (N=292), with relatively even distribution across the age-representation (average 70.52, range 49-102 vs average 69.99, range 45-99, respectively). Further clinical characteristics are summarised in Supplementary Table 2.
Recruitment	Study participants were recruited at time of biopsy (diagnosis) from participating urology clinics within the Gauteng and Limpopo Provinces of South Africa and consented as part of the Southern African Prostate Cancer Study (SAPCS).
Ethics oversight	Study approval was granted by the University of Pretoria Faculty of Health Sciences Research Ethics Committee (HREC #43/2010, with US Federal wide assurance FWA00002567 and IRB00002235 IORG0001762). Genomic interrogation was performed under approval granted by the St. Vincent's Sydney HREC (#SVH/15/227) and established data sharing agreement between the University of Pretoria in South Africa and University of Sydney in Australia.

Note that full information on the approval of the study protocol must also be provided in the manuscript.

Field-specific reporting

Please select the one below that is the best fit for your research. If you are not sure, read the appropriate sections before making your selection.

☒ Life sciences ☐ Behavioural & social sciences ☐ Ecological, evolutionary & environmental sciences

For a reference copy of the document with all sections, see nature.com/documents/nr-reporting-summary-flat.pdf

Life sciences study design

All studies must disclose on these points even when the disclosure is negative.

Sample size	Our sample size of 798 men was based on available recruitment from urology clinics within South Africa. Our sample sizes are the biggest so far for a prostate cancer GWAS study in South Africa, and is comparable to the previous GWAS studies in Sub-Saharan Africa (see Figure 1).
Data exclusions	Samples were excluded during quality control for 1) being a genetic duplicate of another sample (total 8 pairs of duplicates removed), 2) a single patient presenting with prostate metastasis with squamous cell primary carcinoma, 3) if they were missing their age or ISUP grading (for exome-wide association analyses and gene-based analyses), 4) if they were admixed with another ethnicity (a single sample with west African ancestry).
Replication	Study matched replication data is currently not available for exomic data.
Randomization	Not applicable (case-control study)
Blinding	Not applicable (case-control study)

Reporting for specific materials, systems and methods

We require information from authors about some types of materials, experimental systems and methods used in many studies. Here, indicate whether each material, system or method listed is relevant to your study. If you are not sure if a list item applies to your research, read the appropriate section before selecting a response.

Materials & experimental systems

n/a	Involved in the study
☒	☐ Antibodies
☒	☐ Eukaryotic cell lines
☒	☐ Palaeontology and archaeology
☒	☐ Animals and other organisms
☐	☒ Clinical data
☒	☐ Dual use research of concern
☒	☐ Plants

Methods

n/a	Involved in the study
☒	☐ ChIP-seq
☒	☐ Flow cytometry
☒	☐ MRI-based neuroimaging

Clinical data

Policy information about clinical studies

All manuscripts should comply with the ICMJEguidelines for publication of clinical research and a completedCONSORT checklist must be included with all submissions.

Clinical trial registration	Not a clinical trial
Study protocol	Not a clinical trial
Data collection	Initiated in 2008 in South Africa, the Southern African Prostate Cancer Study (SAPCS) aimed to identifying the genetic and non-genetic factors contributing to aggressive disease presentation across the region and associated ancestral disparity. Study participants were sourced from urological clinics within South Africa including, Polokwane Hospital, Tshilidzini Hospital, Pretoria's Steve Biko Academic Hospital and Dr George Mukhari Academic Hospital between 2010 and 2014. As routine PSA testing is not common practice in these regions, study participants presented at the clinic largely as a result of urological complaints. Subjects were reviewed by local urologists, PSA testing performed, and prostate cancer status defined histopathologically by Gleason score. Control samples were those diagnosed with either benign prostatic hyperplasia (BPH) and/or absence of any clinically definable prostate cancer.
Outcomes	Not a clinical trial
